# Atypical Age‐Related Patterns of Neural Signal Complexity in Autism Spectrum Disorder

**DOI:** 10.1002/cns.71173

**Published:** 2026-09-22

**Authors:** Junxia Han, Linya Liang, Rihong Song, Xiaozhou Zhang, Jiayu Liu, Guoqian Jiang, Xiaoli Li

**Affiliations:** ^1^ Beijing Key Laboratory of Learning and Cognition, School of Psychology Capital Normal University Beijing China; ^2^ Tongliao Special Education School Tongliao Inner Mongolia China; ^3^ Dunyi Rehabilitation and Education Center Beijing China; ^4^ School of Electrical Engineering Yanshan University Qinhuangdao China; ^5^ State Key Laboratory of Cognitive Neuroscience and Learning & IDG/McGovern Institute for Brain Research Beijing Normal University Beijing China

**Keywords:** autism spectrum disorder, electroencephalography, multiscale entropy, neural signal complexity, neurodevelopment

## Abstract

**Background:**

Autism spectrum disorder (ASD) is increasingly conceptualized as a neurodevelopmental condition involving atypical organization of large‐scale neural dynamics. While EEG studies have largely focused on spectral power and connectivity, how neural signal complexity is organized across development in ASD remains poorly understood.

**Methods:**

Resting‐state EEG was recorded from 186 children aged 3–11 years, including children with ASD and typically developing (TD) peers. Participants were further categorized into preschool‐aged and school‐aged groups to examine developmental differences. Multiscale entropy (MSE) was computed across 20 temporal scales to characterize neural signal complexity, and group differences, age‐related patterns, and associations with autism symptom severity were examined.

**Results:**

Across the full sample, children with ASD exhibited significantly higher MSE than TD children across brain regions and temporal scales. Group differences were most pronounced in preschool‐aged children, whereas in school‐aged children they were more restricted to lower temporal scales and frontoparietal regions. In TD children, MSE increased robustly with age across scales, while age‐related associations in ASD were more variable and region‐specific. Additionally, individual differences in MSE were associated with autism symptom severity, particularly in frontal and parietal regions.

**Conclusion:**

These findings indicate that ASD is characterized by atypical developmental organization of neural signal complexity across temporal scales, underscoring the importance of a multiscale developmental perspective for understanding neural dynamics in autism.

## Graphical abstract

Using multiscale entropy of resting‐state EEG, children with ASD show globally elevated EEG‐derived neural signal complexity across temporal scales compared with TD peers. These differences are most pronounced early in development and become more region‐ and scale‐specific with age, indicating atypical developmental organization of neural dynamics in ASD.

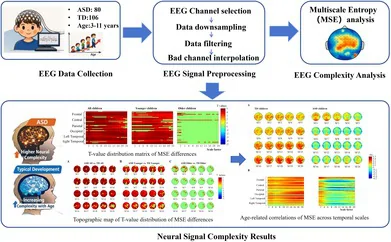

## 1 Introduction

Autism spectrum disorder (ASD) is a heterogeneous neurodevelopmental condition characterized by early‐emerging differences in social communication and restricted or repetitive patterns of behavior [1]. Increasingly, ASD is conceptualized not as a focal deficit localized to specific brain regions, but as a condition associated with atypical development of large‐scale neural systems and their dynamic organization across development [2, 3, 4]. Understanding how neural dynamics unfold across childhood in ASD is therefore critical for elucidating the developmental variability.

Electroencephalography (EEG) provides a noninvasive and developmentally sensitive method for examining neural activity with high temporal resolution [5]. In typically developing children, EEG measures increasingly reveal that neural maturation is accompanied not only by changes in spectral power or connectivity, but also by age‐related increases in signal variability and complexity, reflecting the growing capacity of neural systems to support flexible and integrated information processing [6, 7]. While conventional EEG metrics such as spectral power primarily characterize oscillatory amplitude within specific frequency bands and connectivity measures index interregional synchronization, these approaches may not fully capture the temporal organization of neural activity across multiple timescales [8]. These findings have motivated growing interest in nonlinear measures of neural dynamics that capture properties of brain activity beyond traditional frequency‐based analyses [9].

Multiscale entropy (MSE) is a widely used approach for quantifying the complexity of physiological signals across multiple temporal scales [10]. Rather than indexing randomness per se, MSE characterizes the degree to which neural signals exhibit structured variability across timescales, a property thought to reflect the balance between functional integration and segregation in neural systems [11, 12]. Importantly, by evaluating entropy across coarse‐grained temporal scales, MSE can differentiate meaningful physiological complexity from random signal irregularity, thereby providing complementary information regarding neural efficiency and large‐scale information integration [8, 13]. In the context of development, higher or lower complexity does not necessarily map onto more or less mature brain function; instead, complexity is best understood as reflecting age‐ and region‐specific organization of neural dynamics [7].

A growing number of studies have applied entropy‐based measures to investigate neural dynamics in ASD. Research in infants at elevated familial risk for ASD has shown that early developmental trajectories of EEG complexity differ from those of low‐risk infants, suggesting that atypical neural dynamics may emerge early in development [14]. In older children and adults with ASD, altered patterns of EEG complexity have been reported across multiple cortical regions, including frontal, temporal, and parietal areas, indicating widespread differences in neural signal organization [6, 15]. Given increasing evidence that ASD involves atypical large‐scale neural organization and altered developmental organization of neural systems, MSE may provide a particularly sensitive framework for characterizing developmental alterations in neural dynamics in autism [16]. However, findings across studies have varied in both direction and regional specificity, likely due to differences in age ranges, analytical scales, and sample sizes.

Several important gaps remain in the literature. First, relatively few studies have examined multiscale entropy across a broad pediatric age range within a single framework, limiting our understanding of how neural complexity differs between ASD and typically developing children at different developmental stages. Second, age‐related patterns of MSE in ASD remain incompletely characterized, particularly with respect to whether neural complexity shows similar or altered associations with age compared to typical development. Third, the functional relevance of altered neural complexity remains unclear, as few studies have systematically linked MSE to variability in core autism symptoms, such as sensory processing, communication, and social cognition.

To address these gaps, this study examined resting‐state EEG multiscale entropy in a large sample of children with ASD and age‐matched typically developing controls spanning preschool and school‐age periods. Specifically, we addressed three research questions. First, we asked whether children with ASD differ from typically developing children in EEG complexity across multiple temporal scales and brain regions. Second, we examined whether age‐related patterns of neural complexity differ between groups across early and middle childhood. Third, we investigated whether individual differences in multiscale entropy are associated with variability in autism symptom severity, particularly in domains related to sensory processing, communication, and social cognition. By integrating developmental, neural, and behavioral levels of analysis, this study aims to clarify how atypical neural dynamics relate to developmental heterogeneity in ASD.

## 2 Materials and Methods

### 2.1 Participants

A total of 186 children were recruited, including 80 children with ASD (age range: 3–11 years, *M* = 5.69 ± 2.13 years) and 106 TD children (age range: 3–11 years, *M* = 5.85 ± 1.97 years). ASD diagnoses were made according to DSM‐V criteria. Parents of children with ASD provided relevant clinical information and completed standardized behavioral questionnaires. Children with Rett syndrome, global developmental delay, or other neurological or psychiatric disorders were excluded. TD children were recruited from local kindergartens and primary schools in Beijing and were matched to the ASD group by age and sex. TD participants had no history of neurodevelopmental disorders or family history of ASD. Both groups were evaluated using parent‐reported behavioral questionnaires, including the Autism Behavior Checklist [17], the Social Communication Questionnaire (SCQ) [18], the Social Responsiveness Scale (SRS) [19], and the Clancy Behavior Scale (CABS) [20]. No significant group differences were observed in age or sex. Detailed demographic characteristics and behavioral scores of the participants are summarized in Table 1. The written informed consent was obtained from parents or legal guardians prior to participation.

**TABLE 1:** Participant characteristics.

	ASD children (*n* = 80), mean ± SD (range)	TD children (*n* = 106), mean ± SD (range)	Group difference, *p*
Age (years)	5.69 ± 2.13 (3–11)	5.85 ± 1.97 (3–11)	*t* = −0.523, *p* = 0.600
Male/Female	65/15	79/27	$\chi^{2}$ = 1.178, *p* = 0.278
ABC score	45.36 ± 22.46	8.92 ± 16.69	*p* < 0.001
CABS score	12.32 ± 5.69	NA	
SRS score	89.02 ± 37.17	42.98 ± 14.69	*p* < 0.001
SCQ score	18.25 ± 6.74	5.76 ± 3.22	*p* < 0.001

Abbreviations: ABC, Autism Behavior Checklist; CABS, Clancy Behavior Scale; SCQ, Social Communication Questionnaire; SRS, Social Responsiveness Scale.

### 2.2 EEG Data Collecting and Preprocessing

Eyes‐open Resting‐state EEG data were acquired using a 128‐channel HydroCel Geodesic Sensor Net (Electrical Geodesics Inc., Eugene, OR, USA). Children were seated comfortably and asked to remain still with their eyes open during the approximately 5‐min recording period. Electrode impedances were kept below 50 kΩ, signals were sampled at 1000 Hz, and Cz served as the online reference.

Offline preprocessing was conducted in EEGLAB [21] using MATLAB. Data were down‐sampled to 250 Hz, notch‐filtered at 50 Hz, and band‐pass filtered between 0.5 and 45 Hz. Continuous recordings were segmented into nonoverlapping 4‐s epochs. Artifacts were first identified using an automated algorithm targeting ocular, muscular, and other physiological or nonphysiological noise [22], followed by manual visual inspection to remove residual contamination. Channels with amplitudes exceeding ±200 μV were classified as bad channels, consistent with criteria used in prior studies [23, 24], and were interpolated using spherical spline interpolation implemented in EEGLAB. On average, 2.08 ± 2.64 (mean ± SD) channels were identified as bad and interpolated per participant. After preprocessing, 21.33 ± 4.14 segments for further analysis (ASD: 21.07 ± 4.86 vs. TD: 22.02 ± 4.072). Importantly, the number of retained segments was comparable between groups, and no significant group difference was observed, reducing the likelihood that group differences in MSE were driven by systematic differences in movement‐related artifacts or overall data quality. Given that finer temporal scales may be particularly sensitive to residual myogenic or motion‐related noise, artifact rejection and bad‐channel interpolation procedures were applied prior to MSE estimation to minimize potential contamination. For regional analyses, 61 electrodes were selected from the original 128‐channel montage according to the international 10–10 system to ensure broad coverage of frontal, central, temporal, parietal, and occipital regions, consistent with our prior studies [23].

### 2.3 Multiscale Entropy Analysis

EEG signal complexity was quantified using MSE, a nonlinear metric that characterizes temporal irregularity across multiple time scales by extending conventional sample entropy [13]. Unlike single‐scale entropy measures, MSE incorporates a coarse‐graining procedure to capture scale‐dependent signal dynamics and has been widely applied in the analysis of physiological time series, including EEG.

For each EEG time series, coarse‐grained signals were constructed by partitioning the original signal into nonoverlapping windows of length *s* (scale factor) and computing the average value within each window. This procedure yielded a set of coarse‐grained time series corresponding to different temporal scales. Sample entropy was then calculated for each coarse‐grained series, resulting in entropy values across 20 temporal scales (scale factors 1–20). The embedding dimension (pattern length) was set to *m* = 2, the time delay parameter was set to *t* = 1, and the similarity criterion was defined as *r* = 0.2 times the standard deviation of the time series, consistent with commonly used parameter settings in previous MSE studies [8]. Higher MSE values reflect greater temporal irregularity and increased signal complexity.

### 2.4 Regions of Interest

The 61 EEG electrodes were grouped into six regions of interest (ROIs): frontal, central, parietal, occipital, left temporal, right temporal. For each ROI, mean MSE values were calculated by averaging entropy values across corresponding electrodes.

### 2.5 Statistical Analysis

In order to test the effect of age on EEG complexity, we established a general linear model (GLM) to investigate the multiscale entropy curve with age.

$$Y=\beta_{0}+\beta_{1}\times \mathrm{age}+\beta_{2}\times \text{Diagnosis}+\beta_{3}\times \mathrm{sex}$$

Among them, sex and disease are covariates. Then, the change curve of multiscale entropy of each region of interest with age was further investigated, and the significance level was *p* < 0.05.

In this study, Shapiro–Wilk was used to test whether the multiscale entropy of each group of children in each brain region obeyed normal distribution at all scales. The results show that complexity is normally distributed in all brain regions and at all scales. To detect the differences in brain regions and scales between the autism and normal groups, repeated measure ANOVA was used. Among them, autism status group (ASD vs. TD) and sex (male vs. female) as intersubject variables, scale (SF 1–20) as intra‐subject variables, and age as covariance controlled for the effect of age on complexity. For significant main effects, the next post facto test was conducted using the independent sample *t*‐test. Greenhouse–Geisser corrects the test results. When it comes to multiple comparisons, we use false discovery rate (FDR) for correction. All statistical analysis work in this chapter was performed on SPSS 21.0.

## 3 Results

### 3.1 Intergroup Differences in Multiscale Entropy

**FIGURE 1:**
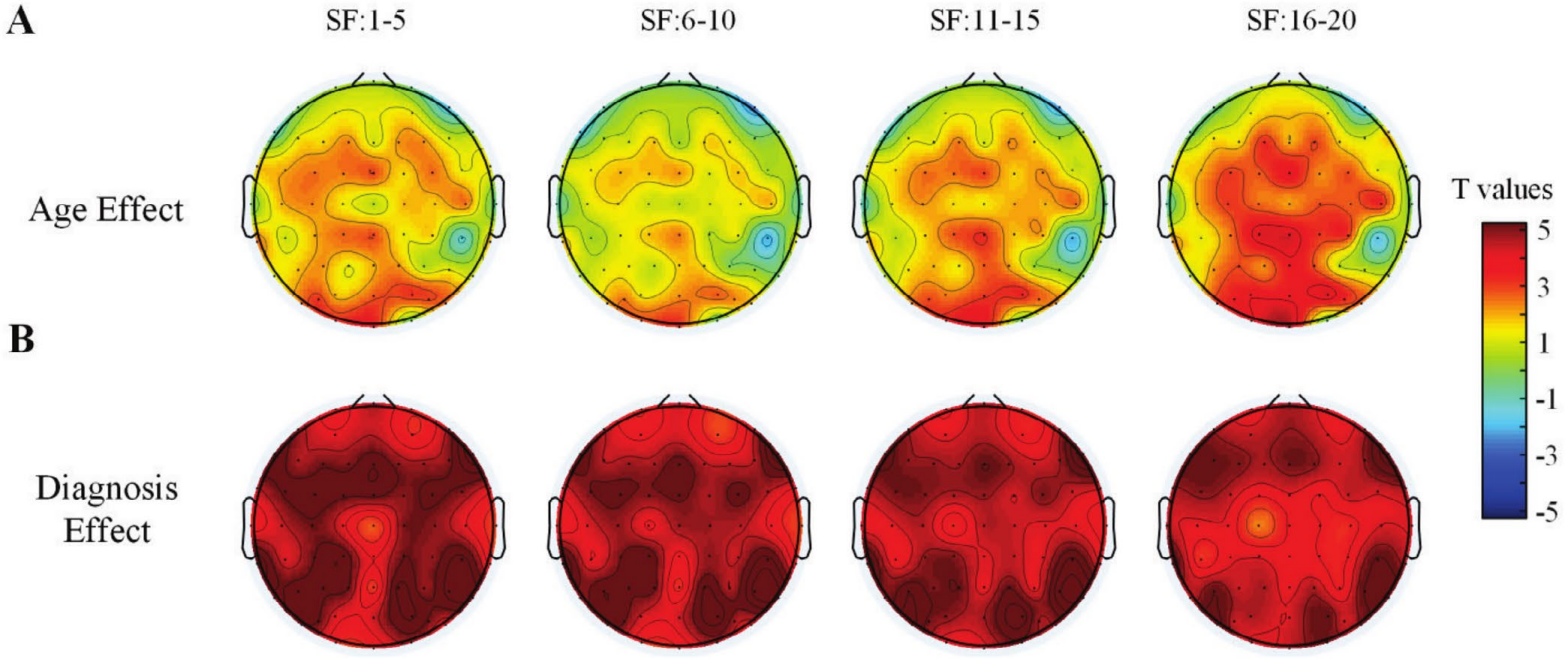
MSE of resting‐state EEG across age in children with ASD and TD children. (A) Main effects of age on MSE at each temporal scale. (B) Main effects of diagnostic group (ASD vs. TD) on MSE at each temporal scale.

The results showed that MSE increased significantly with age across multiple temporal scales in children. This age‐related increase in MSE was observed in both the occipital and central regions (Figure 1A). In addition, significant differences were found between diagnostic groups. As illustrated in Figure 1B, children with ASD exhibited significantly higher MSE values than TD children across all brain‐wide scales.

The ANOVA results for neural complexity are summarized in Table 2. No significant main effect of sex was observed in any brain region; therefore, sex was not included in subsequent post hoc analyses. A significant main effect of diagnostic group (ASD vs. TD) was observed across all examined brain regions, including the frontal, central, parietal, occipital, left temporal, and right temporal regions (all *p* < 0.001). In addition, a significant interaction between diagnostic group and temporal scale was found in each brain region (all *p* < 0.001), indicating that group differences in neural complexity varied as a function of scale. Detailed statistical values for all main effects and interactions are provided in Table 2. Participants were further categorized into preschool‐aged (younger aged from 3 to 6 years) and school‐aged groups (younger aged from 6 to 11 years) to examine developmental differences.

**TABLE 2:** Statistical results of repeated analysis of variance for autistic children.

	All children		Younger children		Older children	
	80 ASD (age range: 3–11 years, age: 5.69 ± 2.13 years) vs. 106 TD (age range: 3–11 years, age: 5.85 ± 1.97 years)		60 ASD (age range: 3 to 6 years, age: 4.61 ± 1.01 years) vs. 76 TD (age range: 3–6 years, age: 4.81 ± 1.07 years)		20 ASD (age range: 6 to 11 years, age: 8.94 ± 1.01 years) vs. 30 TD (age range: 6–11 years, age: 8.49 ± 1.03 years)	
	Group effect (*F*, *p*)	Group × Scale (*F*, *p*)	Group effect (*F*, *p*)	Group × Scale (*F*, *p*)	Group effect (*F*, *p*)	Group × Scale (*F*, *p*)
Frontal	30.489, *p < 0*.001	10.669, *p* < 0.001	26.097, *p* < 0.001	16.540, *p* < 0.001	6.619, 0.013	0.554, 0.564
Central	28.970, *p* < 0.001	11.127, *p* < 0.001	26.594, *p* < 0.001	16.273, *p* < 0.001	3.029, 0.087	0.897, 0.409
Parietal	28.443, *p* < 0.001	9.315, *p* < 0.001	22.017, *p* < 0.001	11.235, *p* < 0.001	5.220, 0.026	1.119, 0.331
Occipital	32.750, *p* < 0.001	9.549, *p* < 0.001	26.255, *p* < 0.001	13.265, *p* < 0.001	6.388, 0.014	0.547, 0.574
Left temporal	39.682, *p* < 0.001	12.625, *p* < 0.001	32.852, *p* < 0.001	18.195, *p* < 0.001	3.510, 0.066	0.107, 0.882
Right temporal	32.953, *p* < 0.001	11.508, *p* < 0.001	24.654, *p* < 0.001	14.315, *p* < 0.001	5.532, 0.022	0.202, 0.786

In the preschool group, a significant main effect of diagnostic group (ASD vs. TD) was observed across all examined brain regions, including the frontal, central, parietal, occipital, left temporal, and right temporal regions (all *p* < 0.001). In addition, a significant interaction between diagnostic group and temporal scale was found in each brain region (all *p* < 0.001), indicating scale‐dependent group differences in neural complexity during early development. In contrast, in the school‐age group, the main effect of diagnostic group was significant in the frontal, parietal, occipital, and right temporal regions (*p* < 0.05), but not in the central or left temporal regions (*p* > 0.05). No significant interaction between diagnostic group and temporal scale was observed in any brain region, suggesting reduced scale‐dependent group differences at later developmental stages.

**FIGURE 2:**
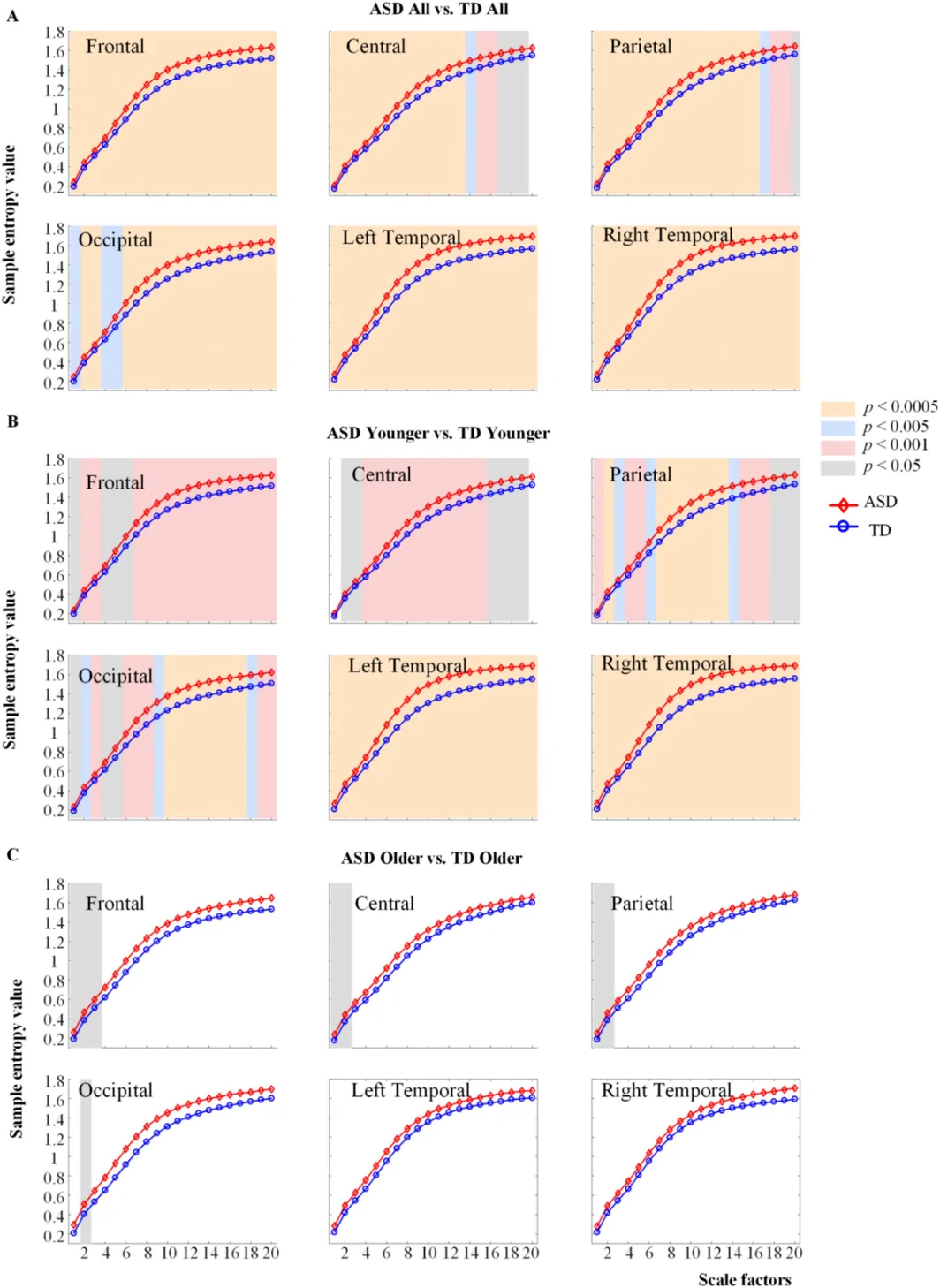
Intergroup differences in multiscale entropy (MSE). (A) Brain regional differences between children with ASD and TD children across the full sample. (B) Brain regional differences between preschool‐aged children with ASD and age‐matched TD children. (C) Brain regional differences between school‐aged children with ASD and age‐matched TD children. Children with ASD are shown in red, and TD children are shown in blue. Different shades indicate ranges of FDR–corrected *p* values.

As shown in Figure 2, independent‐samples *t*‐tests with FDR correction were conducted to examine group differences in multiscale entropy across brain regions. Across the full sample, children with ASD exhibited significantly higher multiscale entropy than TD children in all examined brain regions.

To account for potential age effects, the ASD group was further divided into preschool‐aged and school‐aged subgroups. In the preschool‐aged comparison, children with ASD showed significantly higher multiscale entropy than age‐matched TD children across all brain regions. In contrast, in the school‐aged group, significant group differences were restricted to low temporal scales in the frontal, central, parietal, and occipital regions, with no significant differences observed in other regions or scales.

**FIGURE 3:**
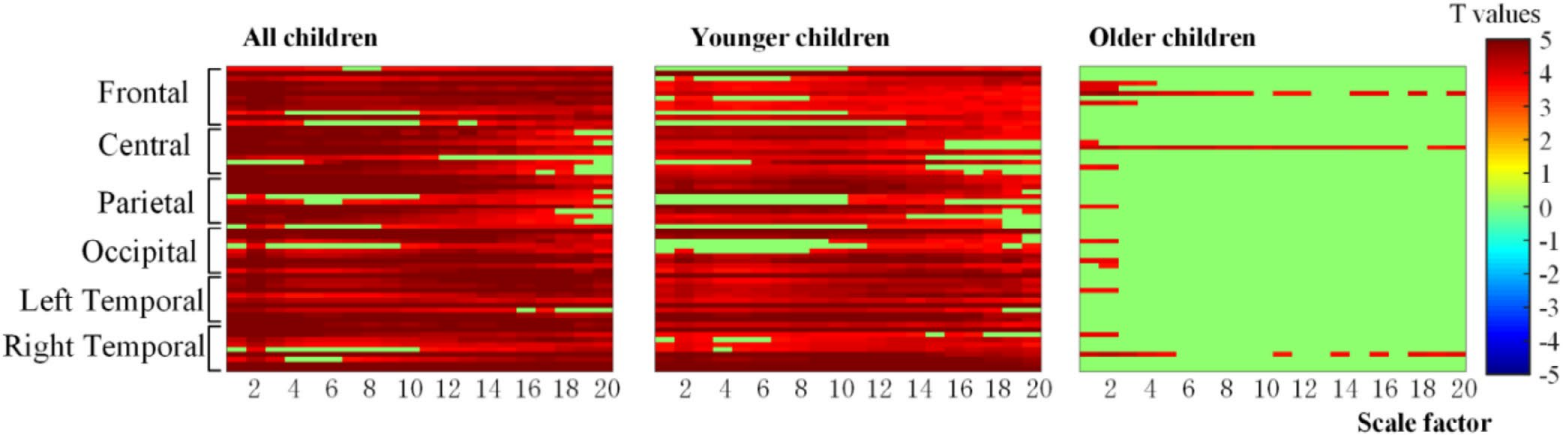
*T*‐value distribution matrix of MSE differences between children with ASD and TD children. The *x*‐axis represents temporal scales (1–20), and the *y*‐axis represents the six brain regions: frontal, central, parietal, occipital, left temporal, and right temporal. Positive values indicate higher MSE in the ASD group relative to the TD group.

To further examine group differences at the channel level, independent‐samples *t‐*tests were conducted on sample entropy values for each EEG channel across multiple temporal scales (Figure 3). Across the full sample, children with ASD exhibited significantly higher sample entropy than TD children across nearly all channels and temporal scales.

When the sample was stratified by age group, preschool‐aged children with ASD continued to show significantly higher sample entropy than age‐matched TD children across all channels and scales. In contrast, in the school‐aged group, group differences were more restricted, with children with ASD showing significantly higher entropy than TD children only at specific temporal scales and channels.

**FIGURE 4:**
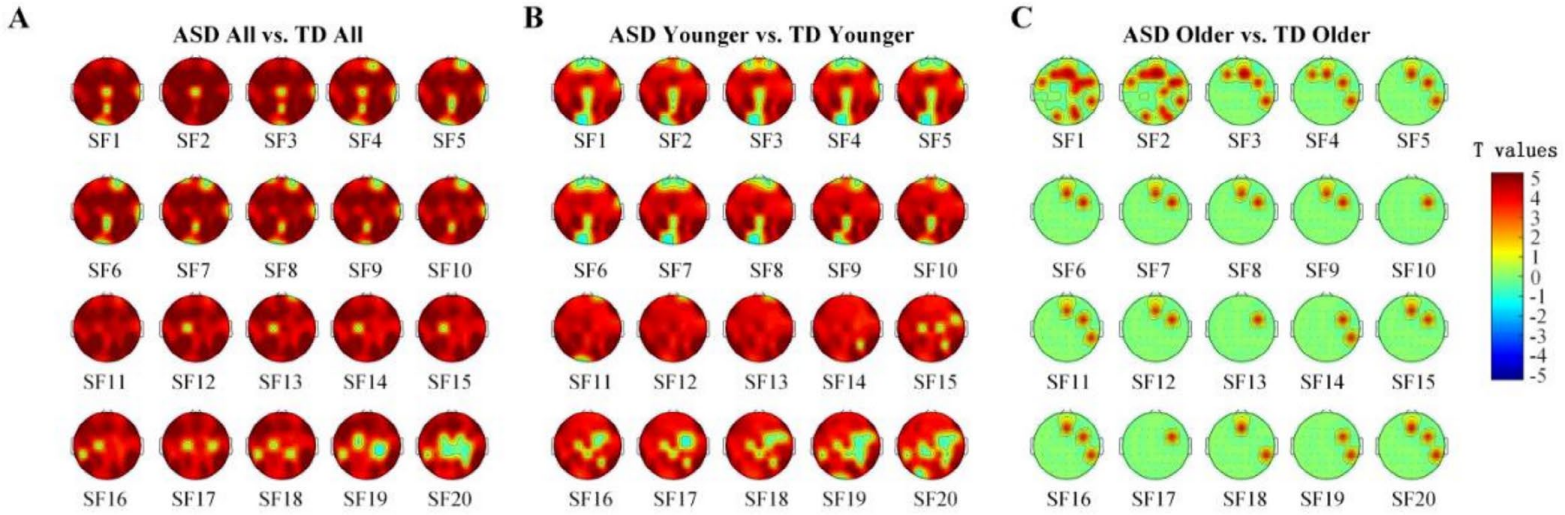
Topographic map of *T*‐value distribution of the difference of multiscale entropy between children with ASD and TD children. (A) ASD all versus TD all, (B) ASD younger versus TD younger, (C) ASD older versus TD older.

As illustrated in Figure 4, topographic maps further revealed the spatial distribution of group differences, indicating that elevated entropy in school‐aged children with ASD was primarily localized to frontal regions.

### 3.2 Multiscale Entropy Changes With Age

**FIGURE 5:**
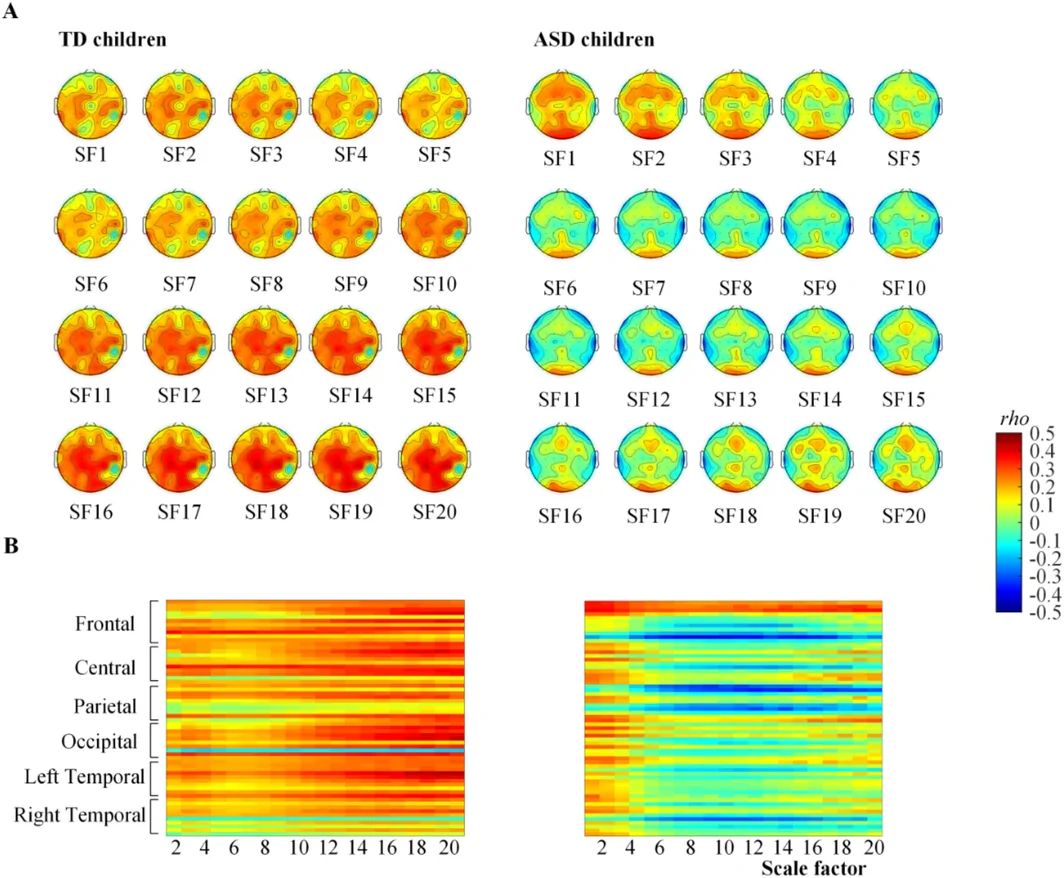
Age‐related correlations of MSE across temporal scales in TD children and children with ASD. (A) Topographic maps illustrating age‐related changes in MSE at different temporal scales for the TD and ASD groups. (B) Matrix representations of age‐related MSE correlations across temporal scales for the TD and ASD groups.

In the TD group, multiscale entropy showed a significant increase with age across all temporal scales, with particularly strong age‐related correlations observed at larger scales (Scales 11–20), as illustrated in the left panels of Figure 5A,B.

In contrast, age‐related changes in multiscale entropy in the ASD group were more region‐ and scale‐specific. Significant associations with age were primarily observed in the occipital and central regions. Specifically, in the ASD group, entropy increased significantly with age mainly in the occipital region at lower temporal scales (Scales 1–5). Conversely, entropy decreased significantly with age in the left and right temporal regions, predominantly at intermediate temporal scales (Scales 6–15).

### 3.3 Associations Between Multiscale Entropy and Behavioral Measures

**FIGURE 6:**
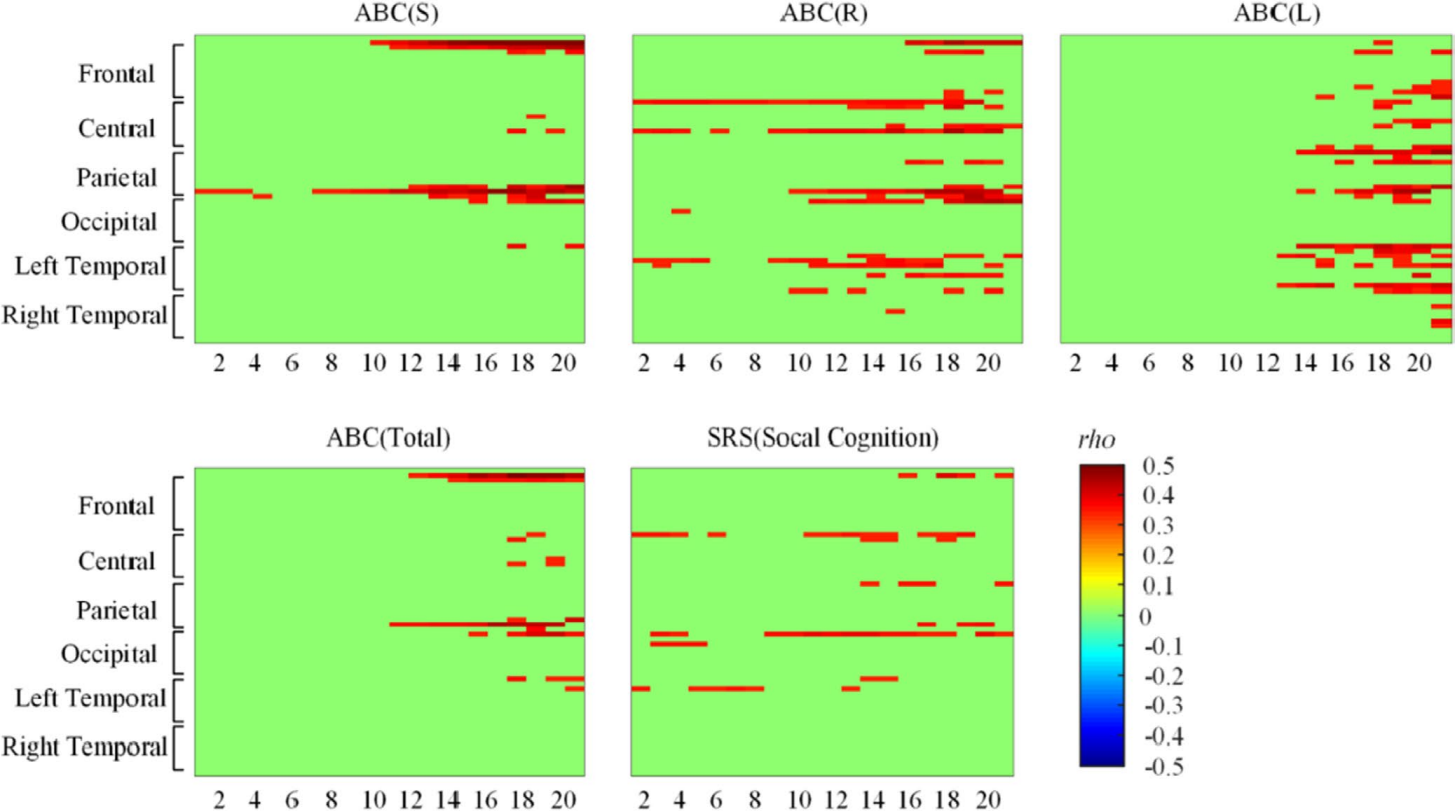
Significant correlations (*p* < 0.05) between autism symptom severity and multiscale entropy across temporal scales and brain regions. Symptom measures include ABC sensory (ABC‐S), communication (ABC‐R), language (ABC‐L), total score (ABC‐Total), and SRS social cognition. Red denotes positive correlations and blue denotes negative correlations.

The severity of autistic symptoms was assessed using the total and subscale scores of the Autism Behavior Checklist (ABC) and the Social Responsiveness Scale (SRS). Spearman correlation analyses were conducted to examine the associations between multiscale entropy values at each scale and channel and the behavioral measures. The results are illustrated in Figure 6.

Significant correlations between entropy values and symptom severity were primarily observed in the frontal and parietal regions. Specifically, multiscale entropy showed significant associations with the sensory, social interaction, and language subscales of the ABC, indicating that neural signal complexity in these regions is closely related to core autistic symptoms.

In addition, correlation analyses with the SRS revealed that entropy values in the central and parietal regions were significantly and positively correlated with social cognition, suggesting that higher neural complexity in these regions may be associated with better social cognitive functioning in children with autism spectrum disorder.

## 4 Discussion

This study examined alterations in neural complexity in children with ASD using MSE analysis of resting‐state EEG, with a focus on group differences, developmental trajectories, and associations with behavioral symptoms. By integrating these dimensions within a multiscale developmental framework, our findings suggest that atypical regulation of neural complexity represents a key neurodevelopmental characteristic of ASD and may help explain how altered brain dynamics relate to behavioral manifestations.

Across the full sample, children with ASD exhibited significantly higher MSE than TD peers across brain regions and temporal scales. While neural complexity has often been associated with greater processing flexibility, converging theoretical and empirical work suggests that this relationship is fundamentally nonlinear, such that excessively high or poorly structured complexity may instead reflect inefficient, noisy, or weakly constrained neural signaling rather than functional advantage [25, 26]. From this perspective, the globally elevated MSE observed in ASD is unlikely to index enhanced neural adaptability at this developmental stage. Rather, it more plausibly reflects atypical organization of intrinsic neural activity across timescales. This interpretation is consistent with prior findings of increased neural variability and reduced signal reliability in autism [27, 28], and extends this literature by demonstrating that such alterations are not confined to a specific temporal window but instead span a broad range of temporal scales captured by MSE.

Further analyses revealed that group differences in MSE were most pronounced in preschool‐aged children, whereas in school‐aged children they became more restricted to lower temporal scales and specific regions, particularly frontal and parietal areas. This pattern suggests that atypical neural complexity may be relatively more salient early in development within the age range studied here, and later manifest in more focal networks supporting executive and social cognitive functions. Such developmental shifts align with cascade models in which early neural atypicalities influence the maturation of higher‐order functional systems [16]. One possible interpretation is that the elevated neural complexity observed in younger children with ASD reflects delayed neurodevelopmental maturation, such that neural dynamics gradually become more similar to TD patterns with increasing age. Alternatively, the attenuation of group differences in school‐aged children may reflect compensatory functional reorganization involving higher‐order cortical systems that support more adaptive neural processing during later development [16, 29]. However, given the cross‐sectional design of the present study, these interpretations remain tentative and should be further evaluated using longitudinal approaches. Consistent with this perspective, frontal and parietal regions, which are key components of executive control and social‐cognitive networks, undergo prolonged developmental refinement across childhood and may therefore be particularly sensitive to early perturbations in neural organization [30, 31].

Importantly, alterations in neural complexity were meaningfully associated with behavioral symptom severity in ASD. Higher MSE values, especially in frontal and parietal regions, were associated with greater impairments in sensory processing, communication, language abilities, and social cognition. These findings suggest that elevated neural complexity is not merely an epiphenomenon of atypical development, but is systematically related to core clinical features of autism. Prior studies have similarly linked increased neural variability to poorer cognitive and social functioning in ASD [27, 32], supporting the interpretation that excessive entropy may index reduced efficiency or reliability of neural information processing. From a systems neuroscience perspective, optimal cognitive functioning depends on a balance between flexibility and stability in neural dynamics [25], a balance that may be disrupted in ASD.

Several limitations should be considered. First, the cross‐sectional design limits inferences regarding individual developmental trajectories; longitudinal studies are necessary to determine whether elevated MSE reflects a stable trait or a dynamically evolving feature of ASD. Second, the exclusive focus on resting‐state EEG does not address how neural complexity may be modulated by task demands, particularly in social or executive contexts. Future studies integrating resting‐state and task‐based paradigms may help clarify the functional significance of altered complexity. Finally, although MSE provides a powerful multiscale characterization of neural dynamics, it is an indirect measure; combining entropy with connectivity, spectral, or excitation–inhibition metrics may yield a more comprehensive account of the underlying neurobiological mechanisms.

In conclusion, by jointly considering group differences, developmental trajectories, and behavioral associations, the present study highlights dysregulated regulation of neural complexity as an important feature of ASD. This multiscale developmental perspective emphasizes that atypical brain function in autism is characterized not simply by increased complexity, but by altered developmental organization of complexity across temporal scales, offering a valuable framework for future longitudinal and mechanistically informed research.

## 5 Conclusion

This study demonstrates that neural signal complexity in autism is characterized by atypical developmental organization across temporal scales rather than a uniform increase in complexity. By adopting a multiscale developmental perspective, our findings highlight the importance of considering age‐dependent trajectories when interpreting neural dynamics in ASD and provide a framework for future longitudinal work linking early neural organization to later behavioral outcomes.

## Author Contributions

**Junxia Han:** data curation, formal analysis, methodology, and writing – original draft, funding acquisition. **Linya Liang:** data curation, methodology. **Rihong Song:** data curation, writing – review and editing. **Xiaozhou Zhang:** data curation, writing – review and editing. **Jiayu Liu:** data curation, methodology. **Guoqian Jiang:** conceptualization, writing – review and editing. **Xiaoli Li:** conceptualization, writing – review and editing.

## Funding

This work was supported by the National Natural Science Foundation of China (62003228).

## Ethics Statement

All participants in this study provided a written informed consent. The study was approved by the ethics committee of the Beijing Normal University (Approval No.: IRB_A_000 92021001).

## Conflicts of Interest

The authors declare no conflicts of interest.

## Data Availability

The data that support the findings of this study are available on request from the corresponding author. The data are not publicly available due to privacy or ethical restrictions.
